# Catatonia and Schizophrenia: Mapping Risk Factors and Treatment Strategies — A Narrative Review Based on a Clinical Case

**DOI:** 10.1192/j.eurpsy.2026.12137

**Published:** 2026-07-31

**Authors:** L. R. Pires, L. Santa Marinha, O. Nombora, A. Marques

**Affiliations:** Psychiatry Department, Unidade Local de Saúde Gaia e Espinho, Vila Nova de Gaia, Portugal

## Abstract

**Introduction:**

Catatonia is a neuropsychiatric syndrome characterized by severe psychomotor disturbance. It may occur in various contexts, mainly associated with schizophrenia (SCZ), affective disorders and non-psychiatric conditions. Despite its clinical significance, catatonia remains underdiagnosed, with significant impact on prognosis.

**Objectives:**

To describe a clinical case of catatonia in a patient with SCZ, and to conduct a narrative review of the literature on this condition, focusing on risk factors and treatment strategies.

**Methods:**

We present a case report and conducted a narrative literature review using relevant scientific bases, including articles focusing on risk factors and treatment of catatonia published over the past 15 years.

**Results:**

39-year-old woman with SCZ presented with stupor, mutism, waxy flexibility, and refusal of food/fluids. A diagnosis of psychotic catatonia was assumed. She had two prior hospitalizations for the same reason, the latest requiring electroconvulsive therapy (ECT). Previously on a long-acting injectable antipsychotic with documented adherence. In the current episode, she was started on intravenous benzodiazepines without benefit, leading to six acute-phase ECT sessions with marked improvement and full resolution of symptoms. No clear cause for decompensation was found. The recurrence of similar episodes raised the hypothesis of an underlying risk factor for this condition.

Literature identifies several risk factors for catatonia, including a prior psychiatric diagnosis, especially SCZ, and previous catatonic episodes. Young age and male sex were also associated. Genetic conditions, including Down, DiGeorge, and Phelan-McDermid syndromes, have been implicated. Associations with neurodevelopmental disorders, including autism spectrum disorder, have been reported, with one study showing a prevalence comparable to other psychiatric conditions. Data on ethnicity are scarce, preventing conclusions about a potential relationship. Finally, a history of emotional trauma has been identified as a risk factor. Early diagnosis is crucial to prevent complications such as dehydration, thromboembolism and rhabdomyolysis. Studies emphasize the importance of treating both the underlying cause and catatonia itself. First-line treatment consists of benzodiazepines, particularly lorazepam, while evidence supporting antipsychotic use is inconclusive. ECT remains the most effective therapy.

**Conclusions:**

Catatonia is mainly associated with psychiatric illness, such as SCZ, and with a history of emotional trauma. Emerging evidence suggests possible associations with genetic conditions and neurodevelopmental disorders, requiring further research. A high index of suspicion is crucial to enable early diagnosis and prevent complications, since its presentation may overlap with other conditions. ECT remains the most effective therapeutic approach in refractory cases.

**Disclosure of Interest:**

None Declared

